# Influence of the Intensive Care Unit Environment on the Reliability of the Montreal Cognitive Assessment

**DOI:** 10.3389/fneur.2019.00734

**Published:** 2019-07-03

**Authors:** Martin Nikolaus Stienen, Olivia Geisseler, Julia Velz, Nicolai Maldaner, Martina Sebök, Noemi Dannecker, Yannick Rothacher, Ladina Schlosser, Nicolas Roydon Smoll, Emanuela Keller, Peter Brugger, Luca Regli

**Affiliations:** ^1^Department of Neurosurgery, University Hospital Zurich, University of Zurich, Zurich, Switzerland; ^2^Clinical Neuroscience Center, University Hospital Zurich, University of Zurich, Zurich, Switzerland; ^3^Neuropsychology Unit, Department of Neurology, University Hospital Zurich, University of Zurich, Zurich, Switzerland; ^4^School of Population and Global Health, University of Melbourne, Carlton, VIC, Australia

**Keywords:** Montreal Cognitive Assessment, intensive care unit, reliability, neuropsychology, cognitive evaluation, neuropsychological assessment, test quality, stroke

## Abstract

**Background:** Neuropsychological screening becomes increasingly important for the evaluation of subarachnoid hemorrhage (SAH) and stroke patients. It is often performed during the surveillance period on the intensive (ICU), while it remains unknown, whether the distraction in this environment influences the results. We aimed to study the reliability of the Montreal Cognitive Assessment (MoCA) in the ICU environment.

**Methods:** Consecutive stable patients with recent brain injury (tumor, trauma, stroke, etc.) were evaluated twice within 36 h using official parallel versions of the MoCA (ΔMoCA). The sequence of assessment was randomized into (a) busy ICU first or (b) quiet office first with subsequent crossover. For repeated MoCA, we determined sequence, period, location effects, and the intraclass correlation coefficient (ICC).

**Results:** *N* = 50 patients were studied [*n* = 30 (60%) male], with a mean age of 57 years. The assessment's sequence [“ICU first” mean ΔMoCA −1.14 (SD 2.34) vs. “Office first” −0.73 (SD 1.52)] did not influence the MoCA (*p* = 0.47). On the 2nd period, participants scored 0.96 points worse (SD 2.01; *p* = 0.001), indicating no MoCA learning effect but a possible difference in parallel versions. There was no location effect (*p* = 0.31) with ΔMoCA between locations (Office minus ICU) of −0.32 (SD 2.21). The ICC for repeated MoCA was 0.87 (95% CI 0.79–0.92).

**Conclusions:** The reliability of the MoCA was excellent, independent from the testing environment being ICU or office. This finding is helpful for patient care and studies investigating the effect of a therapeutic intervention on the neuropsychological outcome after SAH, stroke or traumatic brain injury.

## Introduction

Cognitive assessment is increasingly recommended in patients surviving aneurysmal subarachnoid hemorrhage (aSAH) and is gradually becoming a part of routine clinical practice (1). The high incidence of neuropsychological deficits after aSAH and their decisive impact on long-term recovery are well-described in the literature (2).

Screening of neuropsychological functions to estimate the need for rehabilitation is often already performed during the surveillance period on the intensive care unit (ICU). For this, the Montreal Cognitive Assessment (MoCA) has been identified as first choice among the short but comprehensive instruments (3, 4). It has found entry into national guidelines and the “Outcome and Endpoints” recommendations by the National Institute of Health (NIH)/National Institute of Neurological Disorders and Stroke (NINDS) Common Data Elements (CDE) project (1, 5, 6). Thus, the MoCA is likely to be increasingly applied in the future but it remains unknown, whether the distraction in the ICU environment might bias the results. Furthermore, information on the MoCA test's qualities derives from patients with mild cognitive impairment or Parkinson's disease (7, 8). Especially the MoCA test-retest reliability has never been determined in patients with acutely injured brains (9, 10).

This study set out to determine the test-retest reliability of the MoCA in alert patients with recent brain injury. It also aims to shed light on the question, whether the ICU environment represents a significant bias to MoCA test results.

## Materials and Methods

We prospectively screened all patients suffering from acute brain injury requiring in-hospital treatment at our department between July 2017 and October 2017, e.g., for the (surgical) treatment of intracranial tumors, hemorrhagic or ischemic stroke, hydrocephalus or traumatic brain injury (TBI). The aim was to study a cohort of non-SAH patients, which shares many important characteristics of a typical SAH population, however.

Only alert adult patients (Glasgow Coma Scale ≥ 13) with stable neurological as well as general health status, with sufficient German language skills were enrolled from our regular neurosurgery ward. Patients requiring intensive care were not considered, as it would have been unsafe to test them in a non-ICU environment. We also defined suspected fluctuation of the neurological condition/vigilance, known psychiatric disease with potential influence on the MoCA (e.g., dementia, bipolar disorder), insufficient German language skills or need for sedative medication interfering with the MoCA assessment as further exclusion criteria.

Consenting patients were assessed twice within 36 h. The sequence of assessment was determined by a computerized automatic randomization process to distribute patients into (a) busy ICU first or (b) quiet office first, with subsequent crossover to the other location. Significant fluctuations in the neurological or general health status between both neuropsychological assessments were considered an exclusion criterion.

### Neuropsychological Examination

Full paper and pencil MoCA assessments were performed by neuropsychologists, blinded for health-specific patient details. In order to prevent from learning effects and to reduce false reliability, we used the official German MoCA version for the first assessment (ICU or office), and the additional version 2 for the second assessment (ICU or office), as provided by http://www.mocatest.org. MoCA administration and scoring were based on a standard operating procedure. The same neuropsychologist performed repeated assessments in a single patient to avoid inter-rater reliability issues not attributable to the testing environment.

### Testing Environments

Our Neurocritical Care Unit is a fully equipped 12-bed ICU (room 1: eight beds with equipment for artificial ventilation; room 2: four beds without equipment for artificial ventilation), treating 1,200–1,400 critically ill patients with neurological diseases annually and an occupancy rate of about 90%. In each room, opaque but noise-permeable curtains separate patient boxes to insure some privacy. As for normal clinical routine, curtains were allowed closed during the “ICU assessment.”

The “Office assessment” took place in a quiet office on the ward with closed door. Except for the neuropsychologist and the patient, no additional person was allowed in the room during assessment.

### Statistical Considerations

The null hypothesis was that the MoCA results of the “ICU assessment” do not differ from the MoCA results of the “Office assessment.” The approach to the analysis was similar to that used in the case-crossover design, where the location of assessment (ICU vs. Office) is considered the independent variable. We estimated three key effects: sequence, period (time point), and location of testing. The statistical approaches were detailed previously (10).

The clinical relevance of the difference in the MoCA was appraised referring to the reported minimum clinically important difference (MCID) of two points in both patients with aSAH and Parkinson's disease (8, 9).

The intraclass correlation coefficient (ICC) of repeated MoCA was determined and interpreted according to Cichetti with ICC < 0.40 (poor), 0.40–0.59 (fair), 0.60–0.74 (good), and 0.75–1.00 (excellent) (11).

Given the lack of previous data, no formal sample size calculations were performed. We estimated that including 50 complete datasets would be sufficient (10).

### Ethical Considerations

The Institutional Review Board “Kantonale Ethikkommission Zürich” approved the study (BASEC 2017-00103). All patients gave written informed consent. The study was conducted in accordance with Good Clinical Practice (GCP) guidelines and the 1964 Helsinki declaration and its later amendments.

The study was registered under https://clinicaltrials.gov/ (Identifier NCT03032471) and the methodology with pre-defined statistical plan was published (10).

## Results

**Figure 1:**
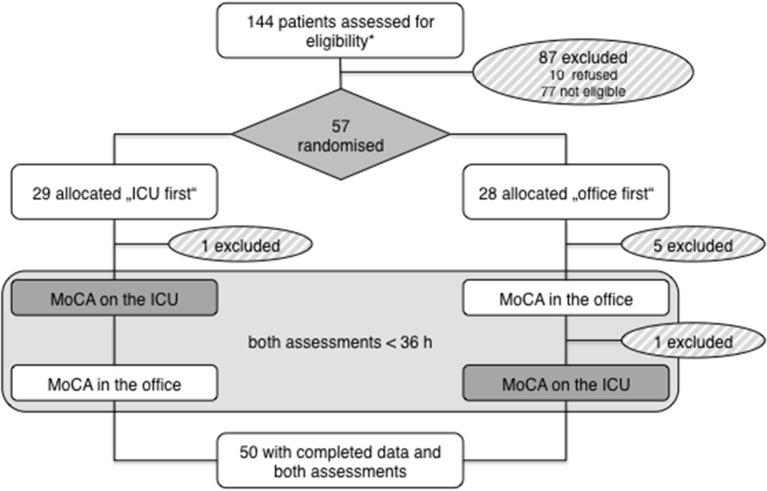
Study profile. *Based on ascertainment log.

A total of *n* = 144 patients were screened, of which *n* = 77 were not eligible and *n* = 10 refused participation. *N* = 57 patients gave consent and were enrolled. *N* = 29 were randomized to the “ICU first” and *n* = 28 to the “Office first” group. During the study, seven patients had to be excluded (six were discharged before the second assessment; one neurologically declined between both assessments). Finally, complete data of *n* = 50 patients was available for analysis (Figure 1).

Patient characteristics from both groups are summarized in Supplementary Table 1. Randomization proved effective to balance for all important baseline parameters, except for patient sex and disease type. Mean MoCA was 24.8 (SD 4.1, range: 14–30; Supplementary Figure 1A) at first and 23.8 (SD 4.6, range 12–30; Supplementary Figure 1B) at second assessment.

### Effects of Sequence, Period, and Location on MoCA Results

The mean difference in the MoCA for patients in the “ICU first” group was −1.14 [standard deviation (SD) 2.34] and for patients in the “Office first” group was −0.73 (SD 1.52; *p* = 0.47), indicating that sequence does not have an effect on the MoCA score.

The mean difference between the MoCA scores at time point 1 and 2 was 0.96 (SD 2.01; *p* = 0.001), indicating that patients scored about 1 point less during the second period.

The mean difference between the MoCA score at the office and ICU was −0.32 (SD 2.21; *p* = 0.31), indicating that location has no effect on the MoCA score (Table 1).

**Table 1:** Reliability measures of the MoCA, taking into account sequence, time point, and location effects.

**Sequence effect**		
ICU first: mean difference −1.14 (SD 2.34)	Office first: mean difference −0.73 (SD 1.52)	*p* = 0.47
**Period effect**		
Mean difference (Time point 1 minus time point 2): 0.96 (SD 2.01)		*p =* 0.001
**Location effect**		
Mean difference (Office minus ICU): −0.32 (SD 2.21)		*p =* 0.31
**Intra-Class Correlation Coefficient (ICC)**		
Individual ICC: 0.87	95% CI: 0.79–0.93	*p <* 0.001
Average ICC: 0.93	95% CI: 0.88–0.96	*p <* 0.001

*CI, confidence interval; ICU, intensive care unit; SD, standard deviation*.

### Intra-Class Correlation Coefficient

The individual ICC was 0.87 (95% CI 0.79–0.93), representing an index for the reliability of different locations for each individual. The average ICC was 0.93 (95% CI 0.88–0.96), representing an index for the reliability of different locations on average (Table 1).

### MoCA Differences ≥2 Points (MCID)

There were 19 (38%) patients in whom the difference in MoCA scores was ≥ 2. It should be noted that the standard deviation of the differences was 2 (similar to the MCID) (9). Knowing that the distribution of differences is normal, and 68% of values of a normal distribution will lie within one standard deviation, that means that ~32% of differences in MoCA scores will be considered to have surpassed the threshold to be considered a clinically meaningful change (MCID).

## Discussion

This study set out to measure the agreement and reliability of the MoCA test when applied to fifty consecutive patients with acutely injured brains in the ICU vs. office environment. Each patient was randomized to being tested in the office or ICU first, served as his/her own control, and was analyzed using crossover design methods. The study demonstrates that there was no location effect on the MoCA results. In general, the reliability of the MoCA was found to be excellent, implying that the difference between the MoCA results of both locations is very small (Table 1). Besides, the study finds that there was no learning effect, but that there may be a difference in the difficulty of the two official parallel versions of the MoCA.

Importantly, the timing of the 1st or 2nd assessment (morning vs. afternoon), the time lag between both assessments, and the severity of headache at time of the assessment was balanced between groups, which is unlikely to have influenced the results (Supplementary Table 1).

Patients with acute brain injury from various causes were chosen as proxy for aSAH patients because of ethical concerns (patient safety), not allowing for aSAH patients to be randomly assigned to assessments on the ICU or in the office. In aSAH patients, bed rest and careful control of hemodynamics, oxygenation and temperature is recommended during the acute phase to minimize the risks for delayed cerebral ischemia. However, the heterogeneous group of patients studied here, many of them having experienced stroke, hydrocephalus and recent brain surgery, resemble well the typical aSAH patient population. A cohort of stable and alert patients with relatively low disease burden was studied (Supplementary Table 1), as it was the aim of the work to investigate the test-retest reliability and medical and/or neurological deterioration between both evaluations would have biased the results. Therefore, it is difficult to extrapolate the findings on the complete population of ICU patients and reliability of the MoCA in severely ill ICU patients might be lower.

Early neuropsychological evaluation finds entry into the management of a broad variety of acute central nervous system disorders, and studying a heterogeneous patient sample allows for generalizing the results to the wider neurosurgical population, including patients suffering from other forms of stroke or TBI.

## Conclusions

In conclusion, the MoCA is a reliable tool when applied to alert patients in an ICU environment. This is an important finding for clinical patient care and studies investigating the effect of a therapeutic intervention on the neuropsychological outcome after aneurysmal subarachnoid hemorrhage, other types of stroke or TBI.

## Data Availability

The datasets used and/or analyzed during the current study are available from the corresponding author on reasonable request.

## Ethics Statement

All procedures performed were in accordance with the ethical standards of the institutional and/or national research committee and with the 1964 Helsinki declaration and its later amendments or comparable ethical standards. The Institutional Review Board Kantonale Ethikkommission Zürich approved the study (BASEC 2017-00103). Informed consent was obtained from all individual participants included in the study.

## Author Contributions

Study conception and design: MNS, NS, EK, PB, and LR. Patient inclusion and clinical data acquisition: MNS, JV, NM, and MS. Acquisition of neuropsychological data: OG, ND, YR, and LS. Study supervision, data analysis, and interpretation: MNS, NS, EK, PB, and LR. Writing of first draft: MNS and NS. All authors editing and revising final draft for intellectual content.

### Conflict of Interest Statement

MNS received research support from Idorsia Pharmaceuticals Ltd., Switzerland for this study. The remaining authors declare that the research was conducted in the absence of any commercial or financial relationships that could be construed as a potential conflict of interest.

## Supplementary Material

Supplementary Figure 1 Histograms of raw MoCA test results. **(A)** Assessment 1. **(B)** Assessment 2.

Supplementary Table 1 Baseline table. *N* = 50 patients were randomized to either MoCA assessment on the ICU or in the office first. Results are expressed as count (percent) or mean (standard deviation). CSF, cerebrospinal fluid; EQ-5D, EuroQol 5D (3L); mRS, modified Rankin Scale; NIHSS, National Institute of Health Stroke Scale; VAS, visual analog scale.

## Abbreviations

aSAH: aneurysmal subarachnoid hemorrhage
CDE: Common Data Elements
CI: confidence interval
CSF: cerebrospinal fluid
EQ-5D: EuroQol 5D (3L)
ICC: intraclass correlation coefficient
ICU: intensive care unit
MCID: minimum clinically important difference
MOCA: Montreal Cognitive Assessment
mRS: modified Rankin Scale
NIH: National Institute of Health
NIHSS: National Institute of Health Stroke Scale
NINDS: National Institute of Neurological Disorders and Stroke
SD: standard deviation
TBI: traumatic brain injury
VAS: visual analog scale.
