# Supplementary figures and images for: Influence of the Intensive Care Unit Environment on the Reliability of the Montreal Cognitive Assessment
Source: Front Neurol. 2019 Jul 3;10:734. doi: 10.3389/fneur.2019.00734 (PMC6617738; doi:10.3389/fneur.2019.00734)

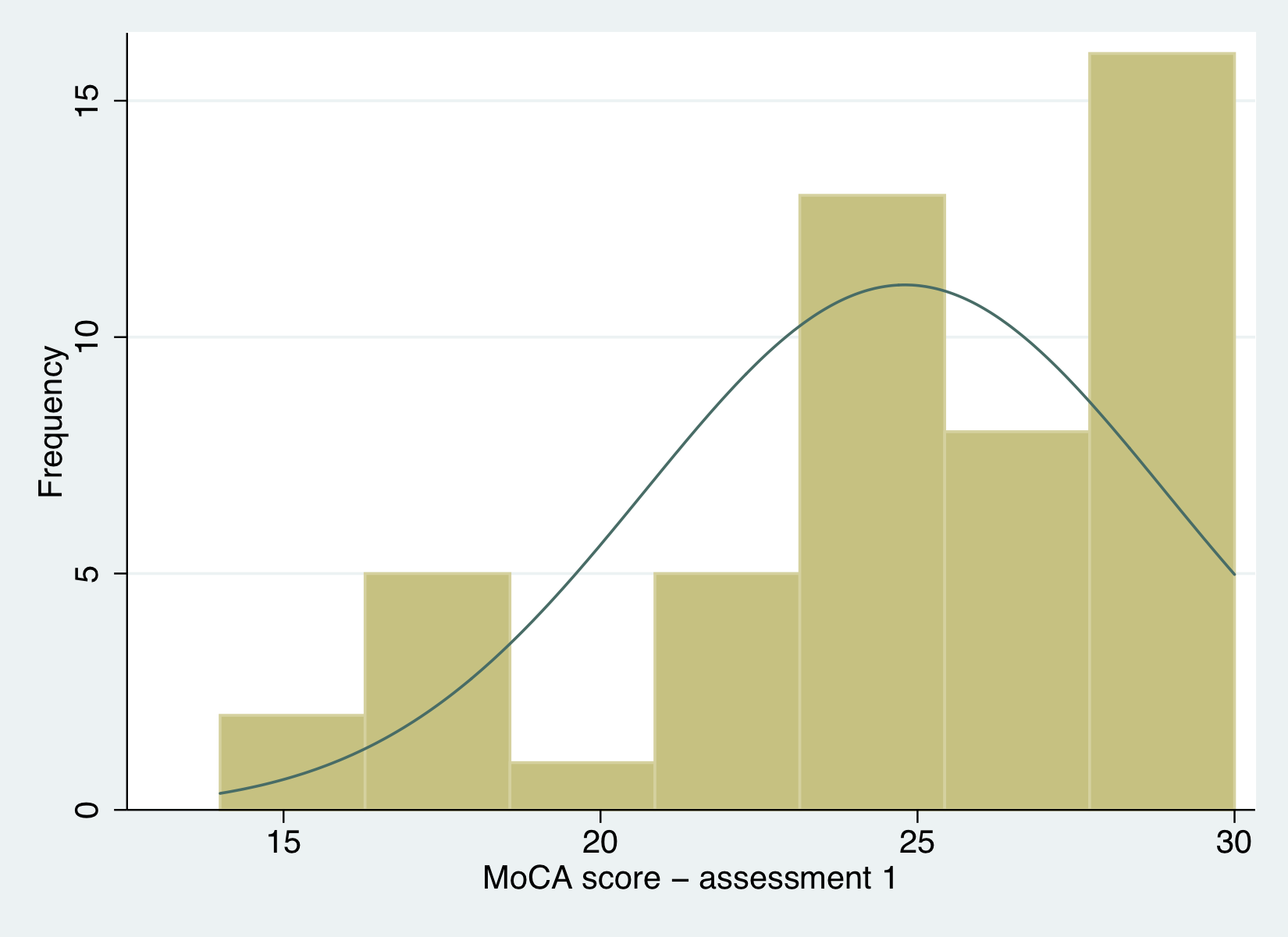

Supplement: Supplementary file 1 [file Image_1.TIFF]

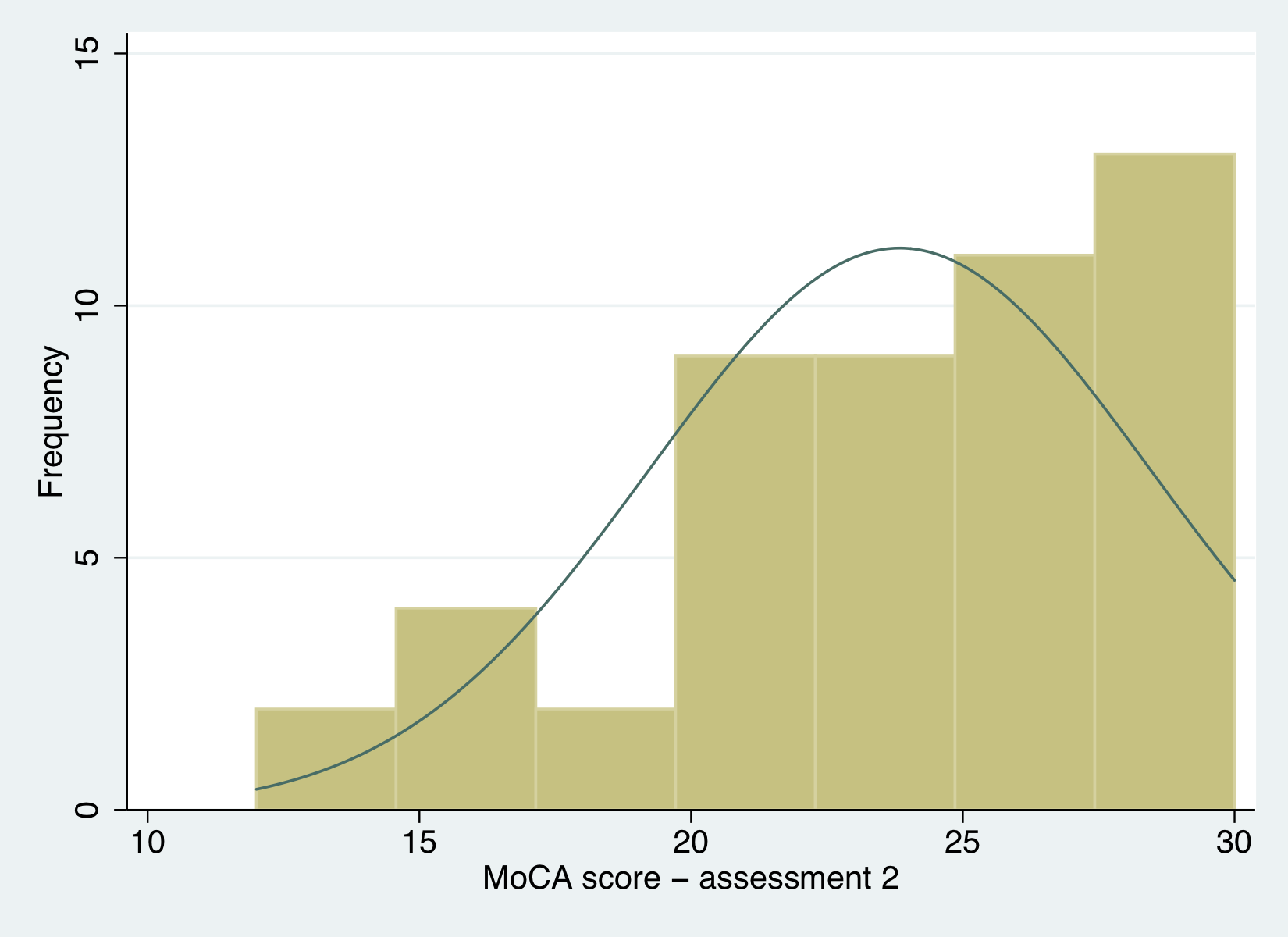

Supplement: Supplementary Figure 1 — Histograms of raw MoCA test results. (A) Assessment 1. (B) Assessment 2. [file Image_2.TIFF]
