# Supplementary material for: Influence of the Intensive Care Unit Environment on the Reliability of the Montreal Cognitive Assessment
Source: Front Neurol. 2019 Jul 3;10:734. doi: 10.3389/fneur.2019.00734 (PMC6617738; doi:10.3389/fneur.2019.00734)
Supplement: Supplementary Table 1 — Baseline table. N = 50 patients were randomized to either MoCA assessment on the ICU or in the office first. Results are expressed as count (percent) or mean (standard deviation). CSF, cerebrospinal fluid; EQ-5D, EuroQol 5D (3L); mRS, modified Rankin Scale; NIHSS, National Institute of Health Stroke Scale; VAS, visual analog scale. [file Table_1.docx]

**Supplementary Table 1:** Baseline table. N=50 patients were randomized to either MoCA assessment on the ICU or in the office first. Results are expressed as count (percent) or mean (standard deviation). CSF = cerebrospinal fluid; EQ-5D = EuroQol 5D (3L); mRS = modified Rankin Scale; NIHSS = National Institute of Health Stroke Scale; VAS = visual analogue scale.

| **Parameter** | **ICU first** | **Office first** | **p-value** |
| --- | --- | --- | --- |
| Age (in years) | 58.7 (18.4) | 54.8 (15.7) | 0.432 |
| Sex  Male  Female | 13 (46.4%)  15 (53.6%) | 17 (77.3%)  5 (22.7%) | 0.027 |
| Race  White  Asian | 28 (100%)  - (0.0%) | 21 (95.5%)  1 (4.5%) | 0.254 |
| Education (in years) | 13.3 (2.6) | 14.0 (3.1) | 0.416 |
| Handedness  Right  Left/forced right/ambidexter | 24 (85.7%)  4 (14.3%) | 20 (90.9%)  1 (9.1%) | 0.575 |
| Disease type  Traumatic brain injury  Brain tumor  Ischemic/neurovascular  CSF-related/infectious/other | 2 (7.1%)  19 (67.9%)  2 (7.1%)  5 (17.9%) | 3 (13.6%)  9 (40.9%)  8 (36.4%)  2 (9.1%) | 0.045 |
| Previous brain surgery  Yes  No | 27 (96.4%)  1 (3.6%) | 19 (86.4%)  3 (13.6%) | 0.193 |
| NIHSS  0 (No deficit)  1 – 2 (Minor deficit)  3 – 4 (Slight deficit)  5 – 6 (Moderate deficit) | 17 (60.7%)  7 (25.0%)  3 (10.7%)  1 (3.6%) | 15 (68.2%)  3 (13.6%)  2 (9.1%)  2 (9.1%) | 0.668 |
| mRS  0 (No symptoms)  1 (No significant disability)  2 (Slight disability)  3 (Moderate disability)  4 (Moderately severe disability) | 1 (3.6%)  16 (57.1%)  9 (32.1%)  2 (7.1%)  - (0.0%) | 4 (18.2%)  11 (50.0%)  4 (18.2%)  2 (9.1%)  1 (4.5%) | 0.287 |
| Z-score EQ-5D index**^a)^**  < -1.0  -1.0 – 1.0 | 6 (21.4%)  22 (78.6%) | 2 (9.1%)  20 (90.9%) | 0.238 |
| EQ-5D VAS  0 – 49  50 – 79  80 – 100 | 9 (32.1%)  9 (32.1%)  10 (35.8%) | 6 (27.3%)  9 (40.9%)  7 (31.8%) | 0.812 |
| Timing of 1^st^ assessment  Morning  Afternoon | 11 (39.3%)  17 (60.7%) | 9 (40.9%)  13 (59.1%) | 0.907 |
| Timing of 2^nd^  assessment  Morning  Afternoon | 11 (39.3%)  17 (60.7%) | 10 (45.5%)  12 (55.5%) | 0.661 |
| Time between both assessments (in hours) | 18.7 (8.3) | 21.4 (6.6) | 0.203 |
| VAS headache at time of  1^st^ assessment  2^nd^ assessment | 15.6 (23.5)  16.0 (23.6) | 17.3 (15.0)  17.1 (16.6) | 0.769  0.849 |
|  | **N=28 (100%)** | **N=22 (100%)** |  |

**^a)^** Z-score adjusted for sex & age, according to normal population reference values.
